# Genome sequencing-based coverage analyses facilitate high-resolution detection of deletions linked to phenotypes of gamma-irradiated wheat mutants

**DOI:** 10.1186/s12864-022-08344-8

**Published:** 2022-02-09

**Authors:** Shoya Komura, Hironobu Jinno, Tatsuya Sonoda, Youko Oono, Hirokazu Handa, Shigeo Takumi, Kentaro Yoshida, Fuminori Kobayashi

**Affiliations:** 1 Graduate School of Agricultural Science, Kobe University, Rokkodai 1-1, Nada, Kobe, 657-8501 Japan; 2 Hokkaido Research Organization, Kitami Agricultural Experiment Station, Yayoi 52, Kunneppucho, Tokorogun, Hokkaido, 099-1496 Japan; 3 National Agriculture and Food Research Organization, Institute of Crop Science, Kannondai 2-1-2, Tsukuba, 305-8518 Japan; 4 Graduate School of Life and Environmental Sciences, Kyoto Prefectural University, 1-5 Shimogamohangi-cho, Sakyo-ku, Kyoto, 606-8522 Japan; 5 Graduate School of Agriculture, Kyoto University, Kitashirakawa Oiwake-cho, Sakyo-ku, Kyoto, 606-8502 Japan

**Keywords:** Hexaploid wheat, Gamma-irradiated mutant, Whole-genome sequencing, Grain hardness, Pre-harvest sprouting tolerance

## Abstract

**Background:**

Gamma-irradiated mutants of *Triticum aestivum* L., hexaploid wheat, provide novel and agriculturally important traits and are used as breeding materials. However, the identification of causative genomic regions of mutant phenotypes is challenging because of the large and complicated genome of hexaploid wheat. Recently, the combined use of high-quality reference genome sequences of common wheat and cost-effective resequencing technologies has made it possible to evaluate genome-wide polymorphisms, even in complex genomes.

**Results:**

To investigate whether the genome sequencing approach can effectively detect structural variations, such as deletions, frequently caused by gamma irradiation, we selected a grain-hardness mutant from the gamma-irradiated population of Japanese elite wheat cultivar “Kitahonami.” The *Hardness* (*Ha*) locus, including the puroindoline protein-encoding genes *Pina-D1* and *Pinb-D1* on the short arm of chromosome 5D, primarily regulates the grain hardness variation in common wheat. We performed short-read genome sequencing of wild-type and grain-hardness mutant plants, and subsequently aligned their short reads to the reference genome of the wheat cultivar “Chinese Spring.” Genome-wide comparisons of depth-of-coverage between wild-type and mutant strains detected ~ 130 Mbp deletion on the short arm of chromosome 5D in the mutant genome. Molecular markers for this deletion were applied to the progeny populations generated by a cross between the wild-type and the mutant. A large deletion in the region including the *Ha* locus was associated with the mutant phenotype, indicating that the genome sequencing is a powerful and efficient approach for detecting a deletion marker of a gamma-irradiated mutant phenotype. In addition, we investigated a pre-harvest sprouting tolerance mutant and identified a 67.8 Mbp deletion on chromosome 3B where *Viviparous-B1* and GRAS family transcription factors are located. Co-dominant markers designed to detect the deletion-polymorphism confirmed the association with low germination rate, leading to pre-harvest sprouting tolerance.

**Conclusions:**

Short read-based genome sequencing of gamma-irradiated mutants facilitates the identification of large deletions linked to mutant phenotypes when combined with segregation analyses in progeny populations. This method allows effective application of mutants with agriculturally important traits in breeding using marker-assisted selection.

**Supplementary Information:**

The online version contains supplementary material available at 10.1186/s12864-022-08344-8.

## Background

Mutagenesis by X-ray and gamma irradiation has been widely used to generate mutants in crops [1, 2]. These irradiated mutants are used as materials for breeding new varieties, linkage map construction, and gene function analysis. In wheat, morphological and physiological mutants, such as of grain shapes and anthesis, are generated by X-ray and gamma irradiation [1]. Mutant plants provide novel and agriculturally useful traits that contribute to wheat breeding as genetic resources. The frequency of generation of mutant phenotypes and mutation types is dependent on the irradiation dose [1, 3]. Gamma irradiation often causes structural variations in translocations and inversions on chromosomes and large deletions with chromosomal breakages [1]. The technique of radiation hybrid mapping was developed based on the random occurrence of large deletions associated with gamma irradiation. This method assesses the presence or absence of chromosome segments using molecular markers and allows the building of high-resolution maps in wheat and its wild relatives [4, 5]. In addition, causal genetic regions of phenotypes of gamma-irradiated wheat mutants have been mapped using simple sequence repeat markers in segregating populations derived from crosses between wild-type and mutants [6].

Genome sequencing technology has allowed genome-wide genotyping and decoding of genome sequences. For example, the combined bulked segregant RNA-sequencing and exome sequencing approaches have been applied to gamma-irradiated maize mutants to successfully identify causative deletions of mutant phenotypes [7]. In contrast, because common wheat (*Triticum aestivum* L.) is a hexaploid species possessing a large and complicated genome (AABBDD), it is challenging to apply the genome sequencing approach to gamma-irradiated wheat mutants. However, high-quality genome sequences of the common wheat cultivar “Chinese Spring” (CS) have been released [8], and the wheat pangenome project provides 10 reference-quality genome assemblies and five scaffold assemblies of common wheat [9]. The presence of the reference genome and the reduction in the cost of genome sequencing allow exploration of genome-wide polymorphisms even in common wheat, unraveling evolutionary history among cultivated wheat and their wild relatives at high resolution [10]. Currently, the application of genome sequencing to common wheat has become more easily accessible. Genome sequencing of gamma irradiation-induced agronomically beneficial mutants can more efficiently identify genomic regions linked to mutant phenotypes than can classical genotyping approaches.

We developed gamma-irradiated mutants of the common wheat cultivar “Kitahonami.” “Kitahonami” is a Japanese winter wheat elite cultivar obtained from a cross between the cultivars “Kitami 72” and “Hokushin.” It is adapted to the northern area of Japan and provides the high-quality soft grains used to produce the Japanese white salted noodle “udon” [11]. A mutant exhibiting useful agronomic traits could potentially help improve “Kitahonami” as well as other wheat cultivars. We have identified a grain hardness mutant and a pre-harvest sprouting (PHS)-tolerant mutant that are expected to be available for further improvement of wheat cultivars.

The grain hardness of wheat is an essential trait in determining grain flour characteristics. The *Hardness* (*Ha*) locus on the short arm of chromosome 5D regulates the grain hardness of common wheat [12]. Allelic differences in two puroindoline genes, *Pina-D1* and *Pinb-D1*, in the *Ha* locus cause variations in grain hardness ranging from soft to hard in common wheat [13, 14]. Reduced expression of *Pina-D1* and *Pinb-D1* genes by RNAi-mediated gene silencing has been reported to increase grain hardness in transgenic common wheat [15, 16], implying that puroindoline genes are required for the formation of soft grains in common wheat.

The moist conditions provided by precipitation cause PHS in common wheat, a phenomenon of immature seed germination in pre-harvest spikes. PHS causes devastating damage to the yield and quality of wheat grains [17–20]. The beginning of the rainy season in Japan coincides with the timing of the wheat harvest. PHS often occurs when the rainy season begins earlier than usual. Therefore, PHS resistance is one of the most essential wheat breeding goals in Japan [21, 22]. PHS is a quantitative trait controlled by multiple genes and is linked to seed dormancy. Several genes associated with seed dormancy and PHS have been identified, including *MOTHER OF FT AND TEL1* (*MFT1*), *TaMKK3-A*, *Viviparous-1* (*Vp-1*), *Qsd1,* and *Tamyb10* [23–28].

In this study, we developed a method to identify genomic regions linked to mutant phenotypes using the grain hardness mutant and the PHS-tolerant mutant. First, whole-genome resequencing was applied to these mutants. To detect gamma irradiation-induced deleted genomic regions, we conducted a sliding window analysis of depth-of-coverage over the chromosomes. Molecular markers were designed based on deletions and applied to the F_2_ populations generated by crosses between wild-type and mutant strains. Finally, we evaluated the depth-of-coverage and window size of sliding window analysis to detect large deletions in gamma-irradiated common wheat. These analyses will provide a good approach for effective breeding methodology using mutants with useful agronomic traits.

## Results

### The hard grain mutant of Japanese elite wheat cultivar “Kitahonami”

**Fig. 1:**
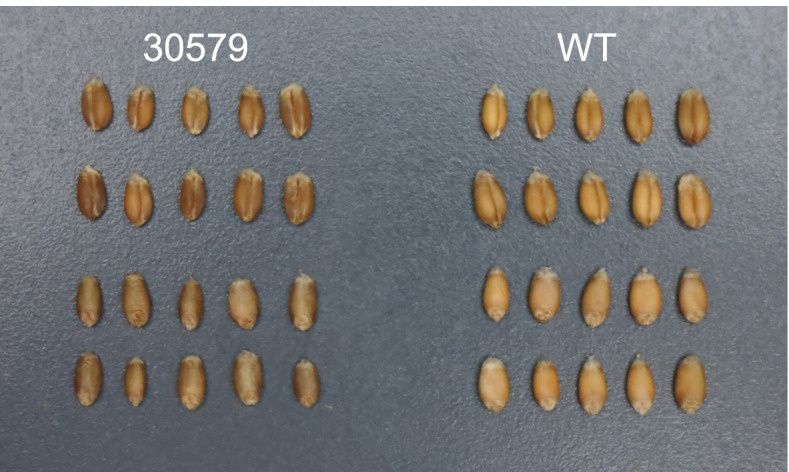
Morphology of the grain hardness mutant “30579” and wild-type (WT) “Kitahonami”. Grains of the mutant “30579” (left) and wild-type “Kitahonami” (right)

To assess the capability of the whole-genome resequencing approach to detect gamma irradiation-induced large deletions in hexaploid wheat, we focused on grain hardness because this trait is regulated by *Pina-D1* and *Pinb-D1* genes, the loss of which changes the grain hardness from soft to hard [15, 16]. Given that “Kitahonami” produces soft grains, the hard grain mutant was assumed to have mutations in the *Pina-D1* and *Pinb-D1* regions on the distal region of the short arm of chromosome 5D. Based on the grain color and the single kernel characterization system (SKCS) value, we obtained the grain hardness mutant “30579” from the gamma-irradiated mutant population of “Kitahonami” (Fig. 1). The SKCS value of the wild-type was 24, whereas that of the grain hardness mutant “30579” was 102 (Table 1). Typically, SKCS values of < 40 and > 70 are regarded as soft and hard grains, respectively. Therefore, grains of mutant “30579” are hard. Grain protein content was also slightly elevated in the mutants (Table 1). In addition, agronomic characteristics of the mutant “30579” were compared with those of the wild-type. Grain weight (kg/ha) and 1000-grain weight of the mutant “30579” were lower than those of the wild-type, indicating that the yield of the mutant was inferior to that of the wild-type.

**Table 1:** Agronomic and quality characteristics of “Kitahonami” and the mutant “30579”

	Kitahonami WT	30579
Agronomic characteristics		
Maturity stage (date)^a^	July 28, 2020	July 27, 2020
Grain weight (kg/ha)	6,870	5,780
1000-grain weight (g)	41.2	30.4
Quality characteristics		
Grain hardness	24	102
Grain protein content (%)	9.4	10.2

^a^Sowing date was September 21, 2019

### Comparative genomics of “Kitahonami” and its hard grain mutant “30579” revealed large deletions induced by gamma irradiation

To identify the causal genome region of the hard grain of the mutant “30579,” genome resequencing of the wild-type “Kitahonami” and the mutant “30579” was performed. In total, 2.3 to 2.9 billion reads were obtained. After removing short reads with poor quality and polymerase chain reaction (PCR) duplicates, 1.9 to 2.3 billion reads were obtained that aligned to the reference genome of CS (Table 2). The average depth-of-coverage was 15.46 for the wild-type and 19.72 for the mutant. The aligned short reads covered more than 94% of the reference genome sequences. The number of single-nucleotide polymorphisms (SNPs) and short indels between “Kitahonami” and CS were 16,096,275 and 732,510, respectively, whereas the number of SNPs and short indels between the mutant “30579” and CS were 24,816,020 and 1,270,877, respectively.

**Table 2:** Summary of alignments and coverages of genome sequencing of wild-type and mutants of “Kitahonami”

Name	Total filtered reads (%)^a^	Aligned reads (%)^b^	Aligned reads after removal of PCR duplicate (%)^c^	Reference bases covered (%)	Average depth-of-coverage
Kitahonami	2,724,430,844 (93.63%)	2,722,445,302 (99.93%)	2,323,699,205 (85.29%)	95.36	15.46
Kitahonami (5x)^d^	881,408,040 (93.62%)	880,765,248 (99.93%)	832,372,258 (94.44%)	93.21	5.51
30579	2,172,729,570 (93.45%)	2,171,397,995 (99.94%)	1,956,235,087 (90.04%)	94.13	19.72
28511	2,393,917,360 (89.52%)	2,391,499,089 (99.90%)	2,159,808,051 (90.22%)	94.87	21.15
28511 (15x)^d^	1,697,754,380 (89.52%)	1,696,038,607 (99.90%)	1,575,442,920 (92.80%)	94.58	15.43
28511 (10x)^d^	1,131,807,308 (89.52%)	1,130,664,237 (99.90%)	1,075,319,384 (95.01%)	94.16	10.53
28511 (5x)^d^	565,916,014 (89.52%)	565,343,556 (99.90%)	550,923,338 (97.35%)	92.76	5.39
28511 (2.5x)^d^	282,949,706 (89.52%)	282,664,292 (99.90%)	278,932,625 (98.58%)	86.31	2.73

^a^An average base quality score per 4 bp >  = 20

The filtered reads rate = Total filtered reads / Total reads × 100

^b^The aligned reads rate = Aligned reads / Total filtered reads × 100

^c^The filtered reads rate = Aligned reads after removing PCR duplicates / Total filtered reads × 100

^d^The sets of read data were used for the simulations. The number in the paratheses indicates approximate depth-of-coverage

**Fig. 2:**
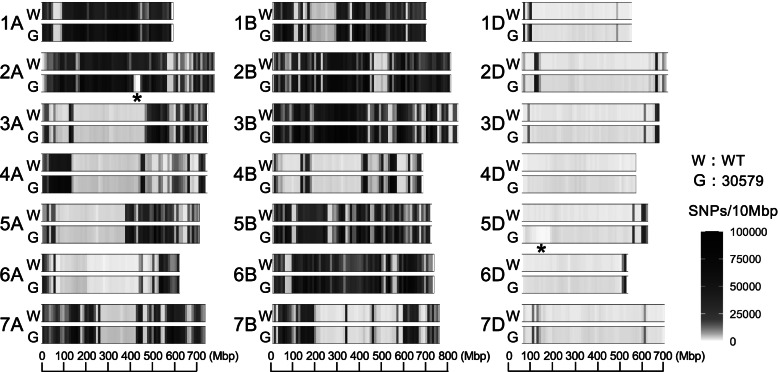
Distribution of SNPs of wheat cultivars “Kitahonami” and the grain hardness mutant “30579” along the chromosomes. SNP density was estimated by counting the number of SNPs per 10 Mbp. The SNP density is displayed as the gradation color from white to black. The higher the density, the blacker it is. In the regions with an asterisk (*), the mutant shows less SNP density compared to the wild-type

The density distribution of SNPs over the chromosome for the mutant “30579” was almost identical to that of the wild-type (Fig. 2). The SNP density for the wild-type and the mutant “30579” was unevenly distributed over the chromosomes. In chromosomes 1A, 2A, 1B, 2B, 3B, 5B, and 6B, “Kitahonami” was genetically divergent from CS, whereas chromosomes 3A, 4A, 6A, 4B, and 7B were genetically close in the two cultivars, particularly in their proximal region. The D genome chromosomes showed less divergence than the other genomes between “Kitahonami” and CS. Less SNP density in the 420–450 Mbp position of chromosome 2A and the short arm of chromosome 5D was uniquely detected in the mutant “30579.”

**Fig. 3:**
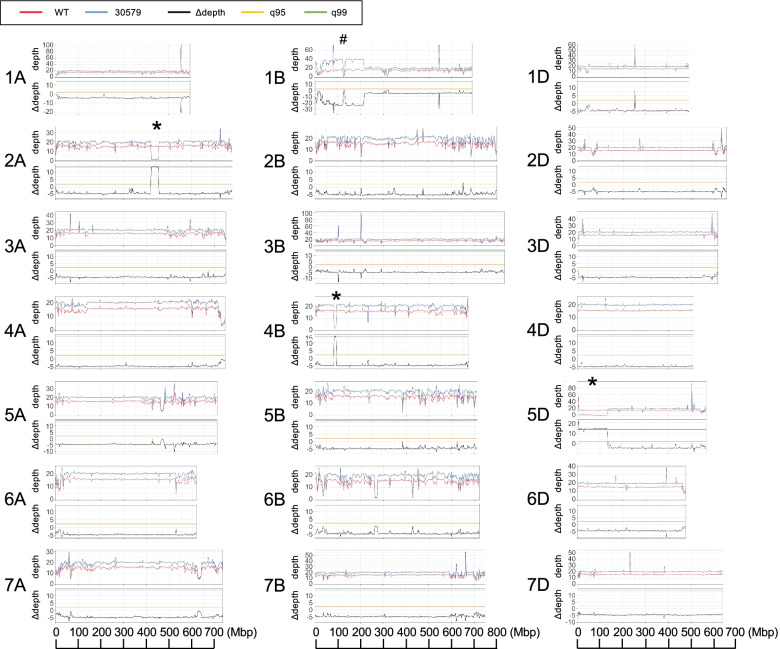
Depth of coverage of sequence reads from wild-type “Kitahonami” and the grain hardness mutant “30579” along the chromosomes. Distribution of the sliding window average of depth-of-coverage and the difference in depth-of-coverage (∆depth) between wild-type “Kitahonami” and the mutant “30579” was visualized along the chromosomes. q95 and q99 indicate 95% and 99% confidence intervals, respectively. The window size was 3 Mbp, and the step size was 1 Mbp. The asterisk (*) indicates a region with a large deletion whereas # indicates a large duplication

Large deletions are often observed in gamma-irradiated mutants [29, 30]. If a large deletion causes *Pina-D1* and *Pinb-D1* to be lost, a decrease in depth-of-coverage in the short arm of chromosome 5D should be uniquely observed in the mutant “30579.” To identify genomic regions where depth-of-coverage uniquely decreased in the mutant, we analyzed the moving average of depth-of-coverage per 3 Mbp over the chromosomes (Fig. 3). Depth-of-coverage in the mutant was close to 0 in 420–450 Mbp position of chromosome 2A, around 90 Mbp position of chromosome 4B, and 0–130 Mbp position of chromosome 5D, indicating that these chromosomal regions were uniquely deleted from the “30579” mutant. The genome regions of the mutant “30579” showed less SNP density than the wild-type (Fig. 2); this was consistent with the deleted regions. Moreover, the distribution of the difference in the depth-of-coverage (∆depth) per 3 Mbp between the wild-type and the mutant “30579” was visualized over the chromosomes (Fig. 3). Peaks beyond the 99% confidence interval were observed at the uniquely deleted regions on chromosomes 2A, 4B, and 5D of the mutant “30579.” Several sharp peaks of ∆depth over 99% confidence interval corresponded to regions with irregularly deep depth-of-coverage, where mapped reads were derived from repetitive sequences. Because the chromosome 5D region with a large deletion corresponded to the genome region containing *Pina-D1* and *Pinb-D1* genes [12], it showed that the hard grain mutant “30579” lost *Pina-D1* and *Pinb-D1* genes.

### Genotyping of mapping population with indel markers confirmed causal regions of hard grains in mutant “30579”

**Fig. 4:**
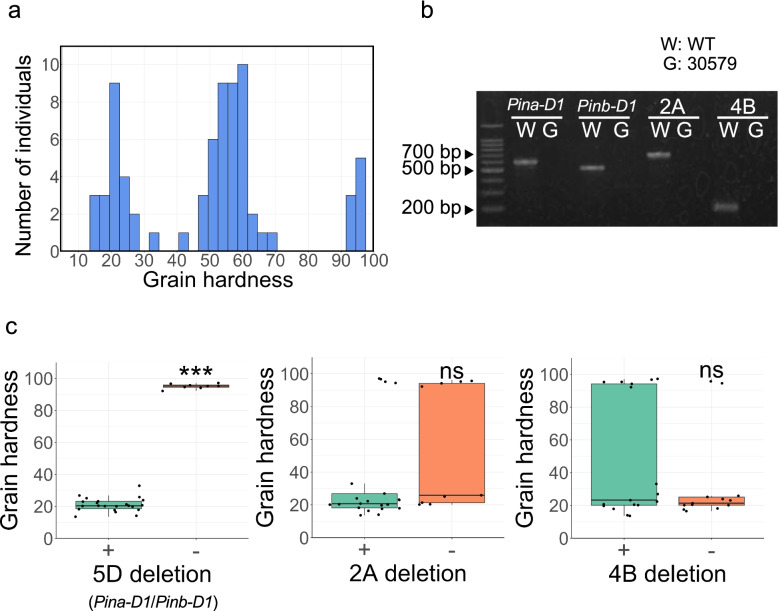
Grain hardness of F_2_ individuals of the cross between wild-type “Kitahonami” and the grain hardness mutant “30579”. **a** The histogram of grain hardness of F_2_ individuals. **b** The results of PCR amplification of the indel markers of 5D (*Pina-D1* and *Pinb-D1*), 2A, and 4B. W and G indicate the wild-type and the mutant, respectively. **c** Relationship between grain hardness and genotypes corresponding to 5D, 2A, and 4B deletions. Boxplots showing the effect of deletion mutations in comparison to wild-type on grain hardness. Dots in the boxplots indicate grain hardness for each sample. Genotypes of 22 soft grain and eight hard grain lines were evaluated based on the indel markers for 5D (*Pina-D1* and *Pinb-D1*), 2A, 4B deletions. A plus sign ( +) indicates the absence of deletion, and a minus sign (–) indicates presence of deletion. Student's *t*-test was performed to test the statistical significance of the differences between the genotypes. Here, *** indicates *P* < 0.01 whereas ns: refers to nonsignificant. The full gel image of **b** is available in Additional file 3: Fig. S4

We developed an F_2_ (*n* = 72) population from a cross between the wild-type and the mutant “30579” to evaluate the segregation of grain hardness. SKCS analysis was conducted to examine the grain hardness of seeds harvested from the F_2_ population. Of the tested lines, 22, 42, and 8 lines showed soft, intermediate, and hard grain phenotypes, respectively (Fig. 4a). To validate which deletions were linked to the hard grain phenotypes, indel markers were designed for each deletion (Fig. 4b). Genotyping of 22 soft grain lines and 8 hard grain lines was performed using indel markers. The indel marker of chromosome 5D was linked to the hard grain phenotypes, whereas the indel markers of chromosomes 2A and 4B were not (Fig. 4c). This result indicated that the large deletion on chromosome 5D caused “Kitahonami” to change phenotype from soft to hard.

### Characteristics of the PHS-tolerant mutant of “Kitahonami”

**Fig. 5:**
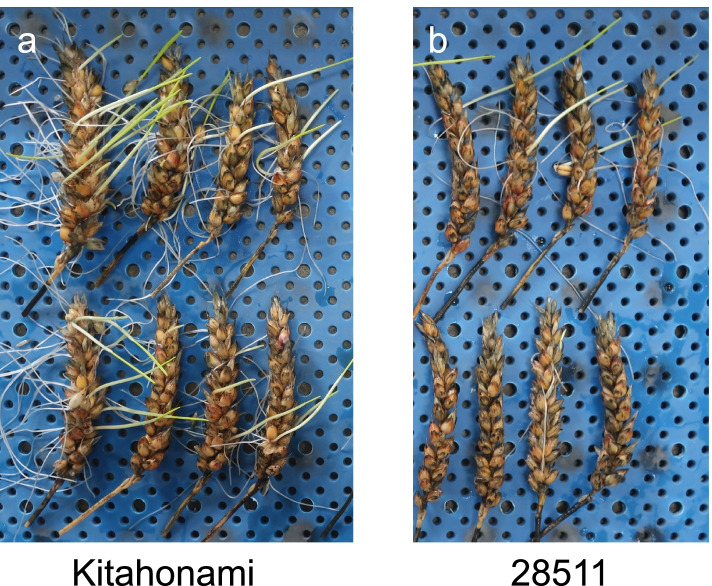
Variation in pre-harvest sprouting tolerance observed on eight spikes of **a** wild-type “Kitahonami” and **b** the mutant “28511”

A PHS-tolerant mutant, called “28511,” was selected from the “Kitahonami” mutant population. The PHS tolerance test showed that the mutant “28511” was more tolerant to PHS than the wild-type (Fig. 5, Table 3). The PHS tolerance of the mutant “28511” was comparable to that of “Kitakei 1831,” which was used as a control variety for PHS tolerance [31]. In the seed dormancy test, the mutant “28511” showed a lower germination rate than the wild-type under both 10 °C and 15 °C conditions in the three seasons (Table 3). The mutant “28511” had a higher germination rate than “Kitakei 1831” in the 2016–2017 season, whereas germination rates of both were similar in the 2017–2018 season. In addition, the agronomic and quality characteristics of the mutant “28511” were evaluated (Table 4). The maturity stage, flour yield, and flour ash content of the mutant “28511” were similar to those of the wild-type. However, grain weight (kg/ha) and 1000-grain weight of the mutant “28511” were inferior to those of the wild-type. Moreover, the mutant “28511” showed higher flour protein content than the wild-type, with discoloration of flour color with respect to darkness (decrease in L* value) and redness (increase in a* value).

**Table 3:** PHS tolerance and seed dormancy tests of wild-type “Kitahonami” and the mutant 28511

Season	Name	PHS tolerance test PHS level (0–5)	Seed dormancy test Germination rate (%)	
			15 °C	10 °C
2016/2017	Kitahonami WT	1.1	64.7	96
	28511	0.3	27.5	83.7
	Kitakei 1838	0	8	54
2017/2018	Kitahonami WT	0.4	83.7	82.4
	28511	0.1	20	45.8
	Kitakei 1838	0.1	18	48
2019/2020	Kitahonami WT	–	72.0	82.0
	28511	–	13.7	26.0

**Table 4:** Agronomic and quality characteristics of wild-type “Kitahonami” and the mutant “28511”

		Kitahonami WT	28511
Agronomic characteristics			
Maturity stage (date)^a^		July 25, 2017	July 26, 2017
Grain weight (kg/ha)		7,470	6,460
1000-grain weight (g)		41.5	36.9
Quality characteristics			
Grain protein content (%)		11.2	13.0
Flour ash content (%)		1.01	1.07
Flour yield (%)		71.0	70.1
Flour color	L*	87.34	86.55
	a*	–0.94	–0.56
	b*	15.43	14.38

^a^Sowing date was September 22, 2016

### Whole-genome resequencing of the PHS-tolerant mutant “28511” identified a large deletion on the long arm of chromosome 3B

To identify the causal region of PHS tolerance of the mutant “28511,” genome sequencing of the mutant was performed using the same method as those for the hard grain mutant “30579.” Of the 2.3 billion qualified reads, 99.9% were successfully aligned to the reference genome of CS (Table 2). The average depth of coverage for the mutant was 21.15. The numbers of SNPs and short indels between the mutant “28511” and CS were 24,508,616 and 1,244,305, respectively. Furthermore, an uneven distribution of SNP density over chromosomes for the mutant “28511” was observed as shown for the wild-type and the mutant “30579” (Additional file 2: Fig. S1).

**Fig. 6:**
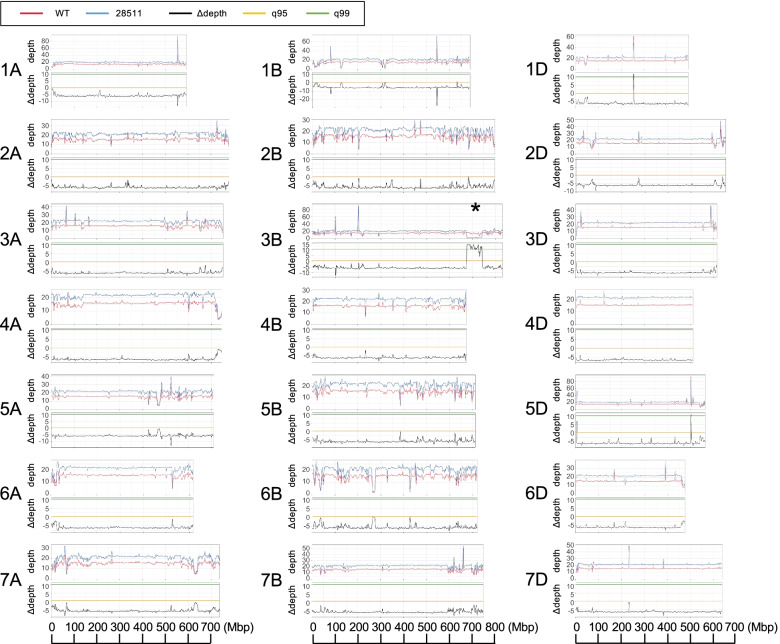
Whole genome sequence depth of coverage for the wild-type “Kitahonami” and the pre-harvest sprouting mutant “28511” along the chromosomes. The distribution of sliding window average of depth-of-coverage and the difference in depth-of-coverage (∆depth) between wild-type “Kitahonami” and the mutant “28511” along the chromosomes, whereas q95 and q99 indicate 95% and 99% confidence intervals, respectively. The window size was 3 Mbp with the step size of 1 Mbp. An asterisk (*) indicates a region with a large deletion

To detect deletions in the mutant “28511,” moving averages of depth-of-coverage per 3 Mbp and ∆depth per 3 Mbp were calculated over the chromosomes. At approximately 700 Mbp position of chromosome 3B, the depth-of-coverage uniquely decreased in the mutant “28511” (Fig. 6). In addition, the ∆depth showed a confidence interval above 99%. The reduced area extended to 67.8 Mbp, indicating that the mutant “28511” had a large deletion in the long arm of chromosome 3B. Such a remarkable reduction in the depth-of-coverage was not observed in other chromosomes.

### Association between the large deletion at chromosome 3B and PHS tolerance

**Fig. 7:**
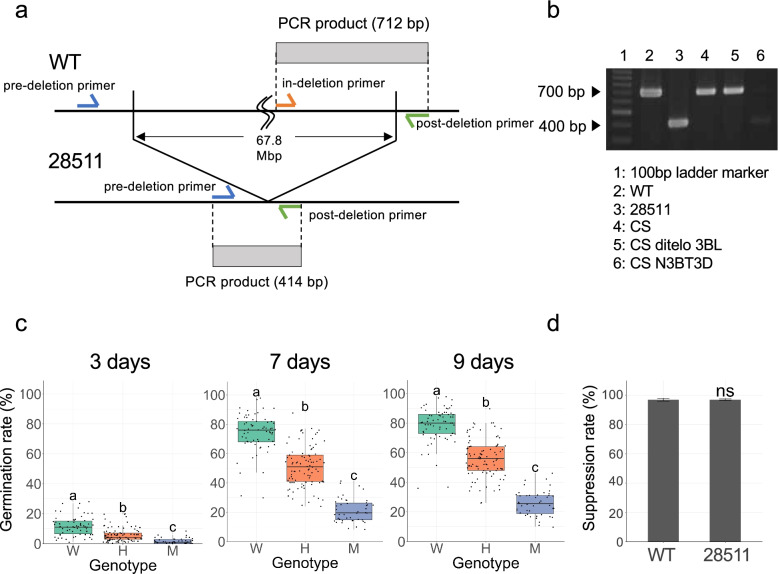
Associations between PHS tolerance and the deletion on chromosome 3B in the F_2_ population from a cross between Kitahonami (WT) and 28511 (PHS mutant). **A** A schematic diagram showing the location of the co-dominant markers designed to detect the deletion on chromosome 3B. A 712 bp PCR fragment is amplified by an “in-deletion” primer, and a “post-deletion” primer in wild-type Kitahonami (WT) whereas a 414 bp PCR fragment amplified by “pre-deletion” primer and “post-deletion” primer is expected in the PHS tolerance mutant 28511. Both fragments are amplified in heterozygous individuals in F_2_ population. **B** Results showing the specificity of the co-dominant markers in the PCR amplification of alleles from WT, “28511”, and the Chinese Spring nulli-tetrasomic line N3BT3D in which chromosome 3B is replaced with chromosome 3D. **C** The seed dormancy test for the F_2_ segregation population of Kitahonami (WT) × the mutant 28511. Boxplots of germination rate for each genotype in 3 days, 7 days, and 9 days are shown. The dots indicate germination rate for each sample. Where W, H, and M are lines carrying homozygous WT, heterozygous and homozygous mutant alleles, respectively. **D** The ABA sensitivity test for root elongation in WT and the mutant, where ns indicates nonsignificant difference based on Student’s *t*-test. The full gel image of **B** is available in Additional file 3: Fig. S5

To validate the large deletion at chromosome 3B, we constructed a co-dominant marker to detect the deletion (Fig. 7a, Additional file 1: Table S1). Primer pairs (pre- and post-deletion primers) were designed outside the deletion boundary. Another primer (in-deletion primer) was designed inside the deletion boundary. In the wild-type, a 712 bp PCR fragment amplified by the in-deletion primer and post-deletion primer was observed, whereas in the mutant, a 414 bp PCR fragment, amplified by pre-deletion primer and post-deletion primer, was observed (Fig. 7b). When the CS nulli-tetrasomic line of nulli-3B tetra-3D, chromosome 3B, which was replaced with chromosome 3D, was used, no PCR fragment was detected, whereas a PCR amplification was detected in the CS ditelosomic 3BL line (ditelo 3BL), where the short arm of chromosome 3BS was lost. These results indicated that this marker was specific to the deletion detected on the long arm of chromosome 3B.

To confirm whether the large deletion was the causal genetic factor of PHS tolerance, we studied the germination rate under three conditions—3, 7, and 9 days at 15 °C after seed sowing—for the F_2_ segregation population from a cross between the wild-type and the mutant “28511.” In addition, we examined the genotype of the F_2_ population using a co-dominant marker to study whether the large deletion was associated with a low germination rate. Under all conditions, the wild-type genotype had a significantly high germination rate, followed by the heterozygous genotype, and the mutant genotype had the lowest germination rate (Fig. 7c). This result indicated that a large deletion was associated with a low germination rate, which could facilitate PHS tolerance of the mutant “28511.”

Because ABA sensitivity, and not ABA levels in seeds, regulates seed dormancy, wheat plants with low ABA sensitivity display reduced seed dormancy, resulting in PHS [32]. To check the ABA sensitivity of the wild-type and the mutant “28511,” the suppression rate of germinated root elongation under exogenous ABA treatment was examined. No significant difference in the suppression between “Kitahonami” and the mutant “28511” was detected (Fig. 7d), implying that they have similar ABA sensitivities.

**Fig. 8:**
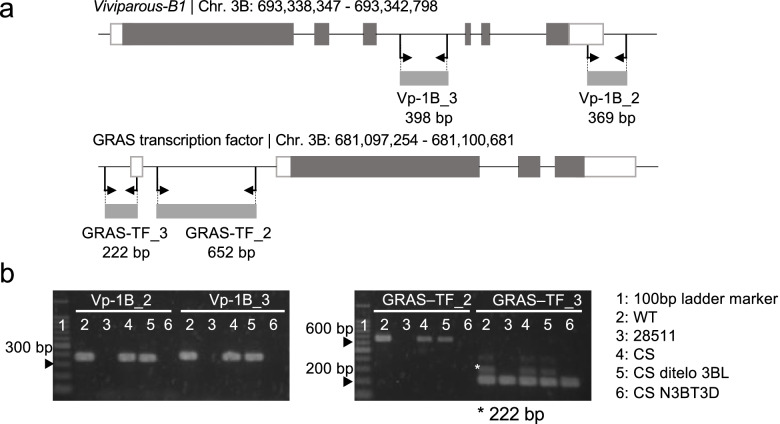
Relationship between PHS tolerance and presence/absence of the candidate genes on chromosome 3D. **a** Primer positions of *Viviparous-B1* and the GRAS transcription factor genes designed to confirm the presence/absence of these genes in “Chinese Spring” (CS) deletion lines at chromosome 3B (Endo and Gill, 1996). **b** The two primer sets were designed to amplify two fragments for each gene. PCR amplifications of *Viviparous-B1* and the GRAS transcription factor gene in wild-type “Kitahonami” (WT), the PHS-tolerant mutant “28511,” CS, the CS 3BL ditelosomic line (CS ditelo 3BL), and the CS nulli-tetrasomic line of nulli-3B tetra-3D (CS N3BT3D). The full gel image of **b** is available in Additional file 3: Fig. S6

*Vp-B1*, associated with PHS tolerance [25, 26], is located on the long arm of chromosome 3B, where a large deletion was detected in the mutant “28511.” In addition, a gene encoding the GRAS family transcription factor was found to be a candidate gene for PHS tolerance in this region. Gibberellic acid (GA) promotes seed germination by inducing the biosynthesis of α-amylase and protease in the aleurone layers [33]. The GRAS family transcription factor *SCARECROW-LIKE 3* (*SCL3*) is a positive regulator of GA signaling in *Arabidopsis thaliana* [34]. We designed two markers for each gene to study whether these genes were absent in the mutant (Fig. 8a). The markers for *Vp-B1* (Vp-1B_2 and Vp-1B_3) showed no amplification in the mutant “28511” and the nulli-3B tetra-3D line. The markers for GRAS family transcription factor (GRAS-TF_2 and GRAS-TF_3) exhibited no amplification in either the mutant “28511” or the nulli-3B tetra-3D line (Fig. 8b). These results confirmed that *Vp-B1* and GRAS family transcription factors were deleted in the mutant “28511.”

### Verification of detection ability in the gamma-irradiated wheat mutant with whole genome sequencing

**Fig. 9:**
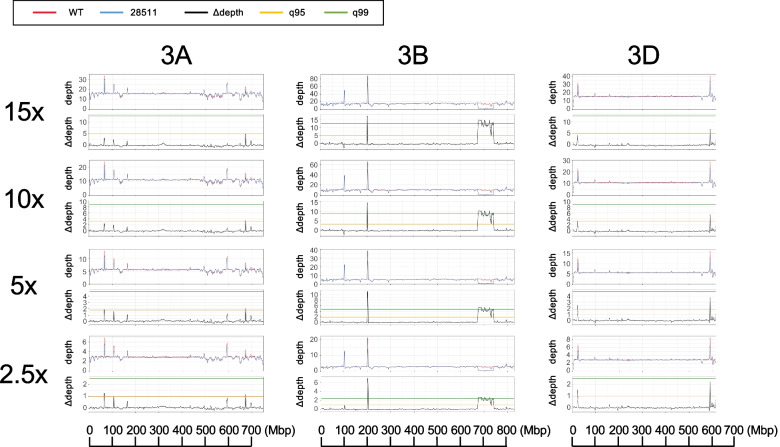
The tests of deletion detection power under different depths of coverage conditions. Distribution of the sliding window average for the depth-of-coverage and the difference in depth-of-coverage (∆depth) between wild-type “Kitahonami” (WT) and the PHS-tolerant mutant “28511” on chromosome 3A, 3B and 3D based on simulated read coverage (15 × , 10 × , 5 × , and 2.5 ×). Chromosome 3BL shows a large deletion responsible for PHS tolerance with ∆depth above q95 and q99 indicate 95% and 99% confidence intervals, respectively

To reduce the resequencing cost, it is essential to know the extent of the depth-of-coverage necessary for detecting large deletions in a gamma-irradiated mutant genome. By subsampling short reads and adjusting the average depth-of-coverage per genome, we evaluated the deletion detection power of the method based on depth-of-coverage, visualized over chromosomes. Four depth-of-coverage conditions, 2.5 × , 5 × , 10 × , and 15 × were tested using short reads from the PHS mutant “28511” and the wild-type (Fig. 9). The distributions of the moving average of depth-of-coverage and ∆depth over the chromosomes were almost identical among all conditions. The 67.8-Mbp deletion on chromosome 3B was significantly detected under all depth-of-coverage conditions, implying that over 2.5 × depth-of-coverage was sufficient to detect a large deletion in the gamma-irradiated wheat mutant.

To clarify how long deletions could be detected under the 5 × depth-of-coverage conditions, the deletion length was simulated by changing the deletion length to 40, 20, 10, 5, 3, and 1 Mbp (Additional file 2: Fig. S2). When the deletion length was 1 Mbp or more, ∆depth was greater than the 99% confidence interval. The length of the detectable deletion was found to depend on the window size of the moving average. If the window size was larger than the length of the targeted deletion, the detectability of the deletion decreased. For example, when the target deletion length was 3 Mbp, the estimated ∆depth for the 3 Mbp or 1 Mbp window size was over 99% confidence interval; however, the estimated ∆depth for a window size of more than 3 Mbp was not. Another peak of ∆depth was present around the 200 Mbp position on the chromosome, which was caused by the repeats derived from transposable elements. Such irregular peaks can be distinguished from deletions by confirming the distributions of both depth-of-coverage and ∆depth.

**Fig. 10:**
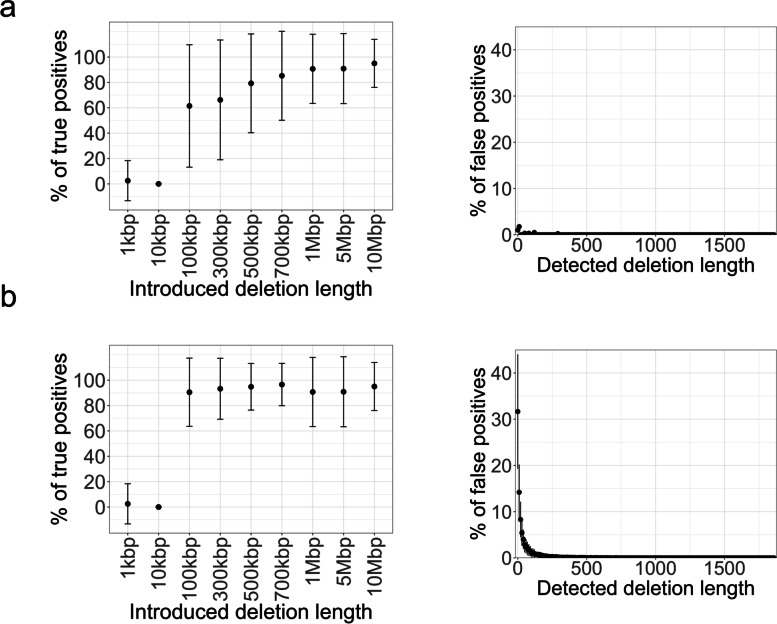
Distributions of true positives (%) and false positives (%). The detection rates of introduced deletions and false-positive ratios when deletions of 1, 10, 100, 300, 500, and 700 kbp, and 1, 5, and 10 Mbp were randomly introduced. The mean and standard deviation after 100 repetitions are shown. Detected deletions were defined as differences in depth-of-coverage (∆depth) between control and deletion introduced sequences exceeding 99% confidence intervals **(a)** or 95% confidence intervals **(b)**

To estimate the sensitivity and specificity of the ∆depth method, deletions ranging from 1 kbp to 10 Mbp were introduced to chromosomes 3A, 3B, and 3D of “Kitahonami”, generating 5 × simulated short reads. The simulated short reads were aligned to chromosomes 3A, 3B, and 3D, and a sliding window analysis of ∆depth was conducted with a 1 Mbp window size and 10 kbp step size to detect the deletions. This process was repeated 100 times to estimate the average sensitivity and specificity of the ∆depth. The average sensitivity values under 95% and 99% confidence intervals were 0.904 ± 0.233 and 0.898 ± 0.231, respectively. The average specificity values for 95% and 99% confidence intervals were 0.997 ± 0.002 and 1.000 ± 0.000, respectively. This result indicated that the ∆depth method had a high sensitivity and specificity, with a large variance in sensitivity. Furthermore, we estimated the distribution of the percentage of true positives (the number of true positives/the number of deletions in an introduced length × 100) and the distribution of the percentage of false positives (the number of false positives in a length/total number of false positives × 100) (Fig. 10). Under the criteria of 95% and 99% confidence intervals, deletion lengths of 100 kbp and 1 Mbp, respectively, were required to detect more than 90% of the introduced deletions. Under the criteria of 99% confidence interval, the percentage of false positives was less than 1.00% if the deletion length was over 20 kbp (Fig. 10a), whereas a > 120 kbp deletion length was required to be less than 1.00% of false positives under the criteria of 95% confidence interval (Fig. 10b).

### Characterization of mutations detected in the gamma-irradiated wheat mutants

**Fig. 11:**
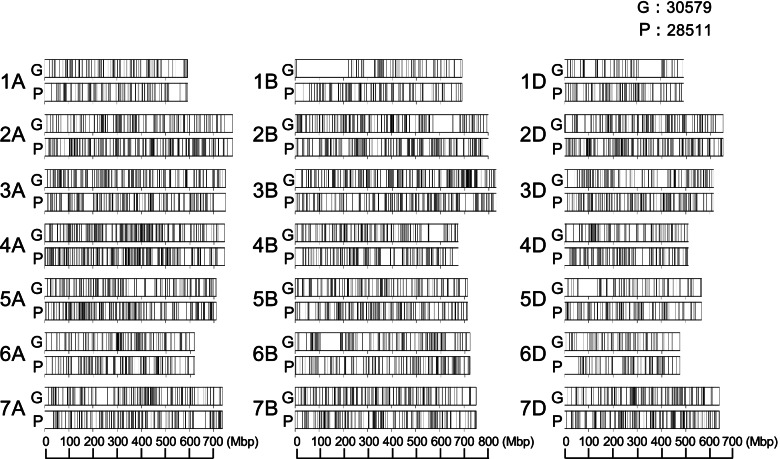
Distribution of SNPs between wild-type “Kitahonami” and the gamma-irradiated mutants along the chromosomes. Each vertical black line corresponds to one SNP location. G indicates the SNP distribution between wild-type “Kitahonami” and the grain hardness mutant “30579” P indicates the SNP distribution between wild-type “Kitahonami” and the PHS-tolerant mutant “28511.”

Nucleotide substitutions and small indels of less than 25 bp, which is the maximum length detected by indel calling using BCFtools [35], were detected in the gamma-irradiated wheat mutants (Table 5, Additional file 1: Table S2). Between the wild-type and the grain hardness mutant “30579,” 2,434 SNPs and 331 small indels were detected. Between the wild-type and the PHS-tolerant mutant “28511,” 2,736 SNPs and 271 small indels were detected. Gamma irradiation is known to cause these SNPs and indels. The ratio of transition and transversion (Ts/Tv ratio) between the wild-type and the mutants was lower than that between the wild-type and CS and was regarded as a natural variation. Over 98% of the SNPs were detected in the intergenic regions. SNPs between the wild-type and the mutants were more randomly distributed over chromosomes compared with SNP density between wild-type “Kitahonami” and CS (Fig. 2, Fig. 11, Additional file 2: Fig. S1). A limited number of natural variations on chromosomes 3A, 4A, 5A, 6A, 4B, and 7B, and all D genome chromosomes were observed between these two cultivars, whereas the putative mutations induced by gamma irradiation covered these chromosomes.

**Table 5:** Comparisons between putative gamma-irradiated and natural SNPs

	Wild-type vs. 30579 (hard grain mutant)	Wild-type vs. 28511 (PHS tolerance mutant)	Wild-type vs. CS
Total	2,434	2,736	16,096,275
Transition	1,579 (64.873)	1,707 (62.390)	11,463,128 (71.216)
Transversion	855 (35.127)	1,029 (37.610)	4,623,419 (28.724)
Ts/Tv ratio	1.847	1.659	2.479
Exon	10 (0.411)	12 (0.439)	106,183 (0.660)
Synonymous	3 (0.123)	2 (0.073)	45,138 (0.280)
Non-synonymous	7 (0.288)	10 (0.365)	61,045 (0.379)
Intron	16 (0.657)	20 (0.731)	247,662 (1.539)
UTRs	4 (0.164)	2 (0.073)	50,368 (0.313)
Intergenic	2,411 (99.055)	2,704 (98.830)	15,796,460 (98.137)
High-impact variants^a^			
Total	0 (0.000)	1 (0.037)	1,648 (0.010)
Non-sense mutations	0 (0.000)	1 (0.037)	830 (0.005)
Start codon lost	0 (0.000)	0 (0.000)	92 (0.001)
Stop codon lost	0 (0.000)	0 (0.000)	276 (0.002)
Splice sites	0 (0.000)	0 (0.000)	454 (0.003)

Percentage is shown in parentheses

^a^Variant types are defined in SnpEff [69]

## Discussion

### Usefulness of genome sequencing-based coverage analysis for deletions and duplications in gamma-irradiated wheat mutants

The present study showed that genome sequencing-based coverage analyses of gamma-irradiated wheat mutants could efficiently detect large deletions. Combined with segregation analyses in the progeny population, deletions linked to mutant phenotypes were successfully identified. The hard grain characteristics require the expression of both PINA and PINB. The confirmation of the deletion of *Pina-D1* and *Pinb-D1* genes at the *Ha* locus in the grain hardness mutant “30579” demonstrated the usefulness of the comparative analysis of depth-of-coverage between wild-type and the mutant. In addition to the detection of large deletions, the depth-of-coverage analysis allowed the detection of large duplications in gamma-irradiated wheat mutants. A putative large duplication was detected on the short arm of chromosome 1B in “30579” (Fig. 3). Depth-of-coverage is used as a parameter for detecting copy number variations [36] and segmental duplications [37]. The approach based on depth-of-coverage fits the detection of deletions and duplications in gamma-irradiated wheat mutants.

The simulation results showed that short sequencing read data corresponding to the 5 × depth-of-coverage over the genome was sufficient to detect 5 Mbp deletions visually. Furthermore, when ∆depth between wild-type and mutant was used, even 1 Mbp deletion could be detected with over 95% confidential interval. The ∆depth approach for deletion detection is more powerful and provides evaluation criteria based on the confidence interval. High-coverage sequencing is not required to detect deletions in gamma-irradiated mutants, thereby reducing the cost of genome sequencing.

Although the ∆depth method showed high specificity and sensitivity, the percentages of true positives and false positives depended on the deletion length. The percentage of true positives decreased and the percentage of false positives increased when the deletion length was shorter than 100 kbp. Therefore, this method is unsuitable for identifying the causal genes of crops with high gene density (Fig. 10). For example, rice has 1 gene per 6.4 kbp [38], and a 100 kbp genomic region is estimated to contain more than 15 genes. Therefore, it is impossible to select a causal candidate gene using the ∆depth method. Bread wheat has a larger genome size and lower gene density than rice. Comparative analysis of multiple wheat genomes showed that the number of genes ranged from 118,734 to 123,075 [9]. Because the whole genome assembly size is 14 Gbp, the gene density per 100 kbp is estimated to be from 0.85 to 0.88. Because the sensitivity and specificity of the ∆depth method are sufficient to detect deletions longer than 100 kbp, a combination of the ∆depth method with segregation analyses in progeny populations potentially narrows down one candidate gene in bread wheat although the gene density is unevenly distributed.

Genome resequencing of the gamma-irradiated rice mutant successfully identified a causal 13 bp-indel for the mutant phenotype [39]. The causal deletion was detected using the HaplotypeCaller tool in the Genome Analysis Toolkit [40], which accurately detects small indels less than 10 bp [41]. In the study of Li et al. [39], structural variations, including large indels, were not detected using the structural variant callers Pindel [42] and Manta [43]. Precision peaks of deletion length for the existing structural variant caller based on short reads are detected between 300 and 500 bp [44]. These callers are not intended to detect over 1 Mbp deletions and are unsuitable for large deletions detected in gamma-irradiated wheat mutants. The ∆depth approach complements the structural variant caller with indel detection.

### Characteristics of deletions and nucleotide mutations in gamma irradiated wheat mutants

This approach is high effective and unique to polyploid wheat with a large genome. Double-stranded DNA breaks induced by gamma irradiation cause large deletions of over 1 Mbp in plants [45, 46]. The inheritance of over 1 Mbp deletion to progeny is often impeded in *A. thaliana* and rice, which are diploid and have a relatively compact genome [29, 45]. The removal of genes involved in viability or gametogenesis is a potential factor in inhibiting the inheritance of large deletions although the mechanisms have not yet been elucidated. In contrast, whole-genome sequencing of the gamma-irradiated wheat mutants identified a ~ 130 Mbp deletion on chromosome 5D and a 67.8 Mbp deletion on chromosome 3B. These large deletions were conservatively inherited by the M_7_ generations (see Methods). The continuous existence of large deletions over generations could be associated with polyploidy. Hexaploid wheat has three homoeologous genes located on each of the A, B, and D genomes. Even when one of the homoeologous genes is lost due to Mbp deletions, the other corresponding homoeologous genes can mitigate deleterious effects on viability. Aneuploid stocks have been established in common wheat. The nullisomic lines, which lost one whole chromosome, were used for genetic research in wheat [47]. In addition, the deletion stocks of common wheat have been generated [48]. Large deletions in these stocks are stably inherited by offspring. Large deletions above 1 Mbp generated by gamma irradiation are highly likely to be stably transmitted to the offspring due to compensation of the homoeologous chromosomes. It is speculated that two double-strand breaks, followed by the removal of the large fragment and subsequent re-ligation at two separated ends of the same chromosome produce a large deletion [45, 49]. Two double-strand breaks could cause large deletions inside the chromosomal ends of the tested gamma-irradiated mutants.

Genome sequencing of the gamma-irradiated wheat mutants detected SNPs and small indels putatively induced by gamma irradiation. The random distribution of SNPs and small indels suggested that gamma irradiation induced these SNPs and indels (Fig. 11). In natural nucleotide variations, a transition is more frequently observed than a transversion, due to differences in the ring structures of nucleobases and amino acid substitution frequencies between the two, although transversions have twice as many nucleotide changes as transitions. The Ts/Tv ratio of putative mutations introduced by gamma irradiation was lower than that of natural variations although transition still occurred more frequently than transversion (Table 5). Because mutations generated by gamma irradiation do not have evolutionary selective constraints on amino acid substitutions, the observed Ts/Tv ratio in gamma irradiation-generated mutations reflect only the differences in the ring structures of nucleobases. Given that the assembly size of the wheat reference genome is 14.5 Gbp [8], the number of SNPs and indels per site were estimated to be ~ 2 × 10^–7^ and ~ 2 × 10^–8^, respectively although these values are potentially underestimated. Given that high-impact SNPs and indels that potentially influence phenotypes were rare in the “Kitahonami” mutants (Table 5), structural variations, such as large deletions and duplications, could be the primary contributors to phenotypic changes in gamma-irradiated mutants of hexaploid wheat.

### Causative gene of PHS tolerance of the “Kitahonami” mutant “28511”

The whole-genome distribution of depth of coverage in the PHS tolerance mutant “28511” demonstrated a 67.8 Mbp large deletion on chromosome 3B (Fig. 6). The co-dominant marker designed for detecting the 67.8 Mbp deletion revealed a statistically significant association between this deletion and PHS tolerance in the F_2_ segregating population (Fig. 7c). Two PHS-related genes, the GRAS family transcription factor (TraesCS3B01G441600) and *Vp-B1*, are located in the deleted region. The deletion of these genes was confirmed by PCR of these genes from the genomic DNA of the PHS-tolerant mutant.

The GRAS family contains a highly conserved C-terminal GRAS domain. *A. thaliana* has 33 genes encoding the GRAS family transcription factor, which includes the DELLA protein family involved in GA signaling in plants [34, 50, 51]. In addition, *Rht-B1* that causes semi-dwarfism in wheat and leads to the wheat green revolution belongs to the GRAS family [52]. TraesCS3B01G441600 is close to *AtSCL1* and *AtPAT1* and is separated from *Rht-B1* and the gene encoding the positive regulator of GA signaling, *AtSCL3* [34] (Additional file 2: Fig. S3). TraesCS3B01G441600 can be categorized into *the AtPAT1* subfamily [53]. *AtPAT1* is involved in phytochrome light signaling rather than GA signaling. *OsCIGR1* and *OsCIGR2*, rice GRAS family transcription factors belonging to *the AtPAT1* subfamily, are induced by exogenous GA in rice suspension culture; however, these are not involved in the mechanisms of α-amylase expression in the aleurone layer [54]. Considering the function of genetically close homologs in *A. thaliana* and rice, TraesCS3B01G441600 is unlikely to be the causative gene of the PHS-tolerant mutant.

*Vp-1* positively contributes to seed maturation, dormancy, and ABA sensitivity in maize, *A. thaliana*, and wheat [25, 55–57], thereby repressing germination. The loss of function of *Vp-1* in maize and *ABI3*, the homolog of *Vp-1*, in *A. thaliana* arrests embryo maturation and decreases ABA sensitivity, resulting in precocious germination [55–57]. The “Kitahonami” mutant “28511” lost the *Vp-1* gene on chromosome 3B and displayed increased PHS tolerance. In addition, the “Kitahonami” mutant “28511” did not show reduced ABA sensitivity. These observations contradict those of diploid plants, maize, and *A. thaliana*. Given that hexaploid wheat has three homoeologous genes, *Vp-A1*, *Vp-B1*, and *Vp-D1*, encoding full-length proteins [58], VP-A1 and VP-D1 could compensate for VP-B1 function, preventing the reduction in ABA sensitivity and blocking precocious germination. Further experiments are required to confirm this hypothesis.

### Effect of gamma-irradiation mutations on agronomic and end-use quality traits

The mutants in this study showed an inferior yield to that of the wild-type (Tables 1 and 4). The decrease in 1000-grain weight of the mutants likely resulted from reduced grain size (Fig. 1). Various mutations were detected in the mutants, including the loss of a considerable number of genes by large deletion. These mutations may directly control the grain size or affect whole plant growth. In the latter case, the impeded whole plant growth also resulted in a decrease in grain size.

Furthermore, grain downsizing may influence the increase in grain protein content: a smaller grain size resulted in a relatively higher protein content. The loss of *Pina-D1* and *Pinb-D1* genes should contribute to a higher protein content in the mutant “30579.” The flour quality of the mutant “28511” was analyzed (Table 4). A slight difference existed between flour ash content and flour yield between the wild-type and “28511,” whereas the flour color of the mutant was discolored. This discoloration was attributed to the increase in grain protein content because a correlation between flour color and protein content was found in a multifamily QTL analysis using “Kitahonami” [59].

Even in hexaploid wheat with compensatory effects by homoeologous genes, mutations, such as large deletions accompanied by loss of a considerable number of genes, are likely to cause severe trait changes such as growth impediment and subsequence yield reduction. Therefore, the causative gene of the target trait should be identified to utilize the mutant more effectively for breeding in the next stage of research.

## Conclusion

In this study, we selected the grain hardness mutant “30579” and the PHS-tolerant mutant “28511” from gamma-irradiated mutants of Japanese elite cultivar “Kitahonami” and performed whole-genome resequencing of these mutant lines. The comparative analysis of depth-of-coverage between the wild-type and the mutants identified ~ 130 Mbp and 67.8 Mbp deletions in the mutants “30579” and “28511,” respectively. These deletions were tightly linked to the mutant phenotypes in the progeny populations generated by crosses between the wild-type and mutant strains. The simulation analyses revealed that 2.5 × of depth-of-coverage was sufficient to detect large deletions in gamma-irradiated hexaploid wheat mutants. These results indicate that short read-based genome sequencing of gamma-irradiated mutants is a cost-effective approach and can be applied to design genetic markers for agriculturally beneficial traits in breeding wheat varieties.

## Methods

### Plant materials

The Japanese elite cultivar “Kitahonami” is a soft winter wheat that provides high-quality flour for Japanese white salted noodles. To generate mutants, the seeds of “Kitahonami” were irradiated with 250 Gy of gamma rays. To screen grain hardness mutants, ~ 1,200 grains were randomly selected from M_2_ mutants. The 26 M_2_ grains with amber-colored grains were selected. Three individuals showing hard grain characteristics that were estimated by the SKCS analysis were selected from the 26 individuals and were regarded as three lines. One line was selected from the three lines of M_4_. The M_7_ mutant line, called “30579,” was obtained by repeated selfing of the selected line. For the segregation analysis, F_2_ progenies were generated from a cross between “Kitahonami” and “30579” (M_7_).

To screen for PHS-tolerant mutants, M_2_ mutant seeds were randomly selected for M_3_ mutants. In the fields of Kitami Agricultural Experiment Station, Kunneppucho, Hokkaido, Japan, 5,000 M_4_ mutants were grown. After flowering, 1,500 spikes were harvested at the maturing stage, sprayed with water in the morning and evening, and incubated at 15 °C for 7 days. Seven spikes with low germination and rooting were selected as the PHS-tolerant candidate lines. After M_5_ generations, we performed PHS tolerance and seed dormancy tests on every generation, and selected candidate lines showing stronger PHS tolerance and seed dormancy than the wild-type. In M_7_, one candidate line showing stronger PHS tolerance and seed dormancy than the wild-type was selected and named “28511” mutant line. For the segregation analysis, F_2_ progenies were generated from a cross between “Kitahonami” and “28511” (M_7_).

To compare the PHS-tolerant level and seed dormancy, “Kitakei 1838,” known as a PHS high-tolerant variety, was used as a control in PHS tolerance and seed dormancy tests. “Kitakei 1838” was developed from the pedigree W148-59–8 (OW104)/98046//99015 through a collaboration between Hokuren Agricultural Research Institute and Kitami Agricultural Experiment Station, and has been used in the breeding program as a PHS-tolerant control [31]. The wheat variety “OW104” in this pedigree was developed as a transgressive highly tolerant line [60].

Wheat aneuploid lines, nulli-3B tetra-3D (N3BT3D) line and the ditelosomic 3BL line of CS [61, 62], were used to examine the 3B-specificity of the primer sets as negative and positive controls, respectively.

### Tests of grain hardness, PHS tolerance, seed dormancy, and ABA sensitivity

Grain hardness was evaluated using SKCS 4100 (Perten, Stockholm, Sweden). The SKCS hardness index was obtained by crushing a sample with at least 50 kernels each from the wild-type and the grain hardness mutant “30579.”

To test PHS tolerance, 10 spikes were detached during the maturity period after flowering, incubated on trays at 15 °C, and sprayed with water in the morning and evening. After 10 days, the spikes were scored on a scale from 0 (no visually sprouting kernels) to 5 (almost every kernel had sprouted). The sprouting score was calculated by counting the number of sprouted kernels and multiplying it by 0.5. If 10 or more sprouted kernels were found, the score was 5. The PHS level was calculated as the average sprouting score among the spikes. The tests were performed annually for two cropping seasons 2016–2017 and 2017–2018 using 10 detached spikes of the season.

For the seed dormancy test, 50 seeds, 7 days after ripening, were incubated in a petri dish at 10 °C or 15 °C. The germination rate was calculated based on the number of germinated seeds after 3, 5, 7, or 9 days. A series of tests was conducted annually for three cropping seasons 2016–2017, 2017–2018, and 2019–2020 using the collected kernels of the season.

To test ABA sensitivity, a bioassay for ABA responsiveness was performed according to the protocol described by Iehisa et al. [63]. Ten seeds of the wild-type or the PHS-tolerant mutant “28511” were treated with running water for 6 h. The seeds were incubated on a wet filter paper in a petri dish at 4 °C under dark conditions overnight and subsequently incubated at 23 °C for 20 h. Two petri dishes with filter paper were prepared. Six milliliters of 20 µM ABA was added to one of the petri dishes; the same volume of ultrapure water was added to the other as a control. After measuring the root length of the germinated seeds, five germinated seeds were placed in each petri dish. The root length of each seed was measured 48 h later. The elongated root length was defined as the difference in the root length between 0 and 48 h. The suppression rate of root elongation was calculated as: elongated root length in ultrapure water–(elongated root length in ultrapure water–elongated root length in ABA)/elongated root length in ultrapure water. Three independent bioassays were performed for the wild-type and PHS-tolerant mutant “28511.”

### Examination of agronomic traits

Agronomic traits of the mutants “30579” and “28511” were examined in the cropping seasons 2019–2020 and 2016–2017, respectively. The experimental field was located at the Kitami Agricultural Experiment Station (lat. 43.7 N and long. 143.7 E.). The maturity stage was recorded when the grains had a 40% moisture content. Grain samples harvested from field trials were subjected to the following analyses [59]: grain weight per 10 ha and 1,000-grain weight were calculated using fully ripened grains, and grain protein content was measured using near-infrared spectroscopy with an Infratec NOVA instrument (FOSS, Hilleroed, Denmark) and adjusted to a moisture content of 13.5%. The grain ash content was determined by combustion at 600 °C for 4 h. The flour yield was calculated as the percentage of total flour weight (A flour + B flour) relative to the sample weight (A flour + B flour + bran). Flour efficiency was calculated as the percentage of A flour weight relative to the total flour weight (A flour + B flour). The A flour was subjected to an additional sieving step using a stainless steel 180 μm testing sieve (Tokyo Screen Co. Ltd., Japan). Sieved A flour was evaluated for color values using a ZE-6000 m (Nihon Denshioku, Japan) based on three-dimensional color values with the following rating scale: L* value for whiteness (100: white, 0: black), a* value for red-green chromaticity (+ 60: red, − 60: green), and b* value for yellowness (+ 60: yellow, − 60: blue). A 6-g flour sample was combined with 10 mL of distilled water to form a paste, which was mixed well without bubbling. Flour paste was poured into a Petri dish for ZE-6000 analysis, and the three color parameters were measured with the illuminant C and angle of 2° settings.

### Extraction of total DNA and genome sequencing

The total DNA was extracted using the hexadecyltrimethylammonium bromide (CTAB) method from the leaves of wild-type and mutant plants of “Kitahonami.” After RNase treatment, a DNA library for 100-bp paired-end DNA sequencing of the wild-type was built using the TruSeq DNA library preparation kit (Illumina, San Diego, CA, USA). DNA sequencing was performed on an Illumina HiSeq-X sequencer. PCR-free DNA libraries for 150-bp paired-end DNA sequencing of the mutants were constructed using the TruSeq DNA library preparation kit (Illumina). DNA sequencing of the mutants was performed using a NovaSeq sequencer. The sequencing reads were deposited in the database of the Komugi GSP (Genome Sequencing Program) (https://komugigsp.dna.affrc.go.jp/research/download/index.html).

### Quality control, alignments, and SNP calling

The quality of the sequencing reads was checked using the FASTQC ver. 0.11.7 [64]. Trimmomatic version 0.33 [65] was used to exclude short reads with an average minimum Phred quality score per 4 bp less than 20 and a length of less than 40 bp. The filtered paired-end reads were aligned to the reference genome of CS version 1.0 [8] using BWA version 0.7.17 [66] with default options. Paired-end reads putatively generated by PCR duplications were removed using SAMtools version 1.9 [67]. The average number of aligned reads and genome coverage of short reads over the reference genome were calculated using BBMap version 37.77 [68]. SNP and indel calling were conducted using BCFtools version 1.9 [35]. The command lines used for SNP and indel calling are described in Additional file 1: Table S3. Annotations of SNPs and indels were conducted using SnpEff version 4.3t [69].

### Detection of large deletions induced by gamma irradiation

The depth-of-coverage for each genome position was estimated using SAMtools version 1.9 [67]. To detect deletions from the distribution of depth-of-coverage over chromosomes, the moving average of depth-of-coverage was calculated with a window size of 3 Mbp and a step size of 1 Mbp using Python version 3.7.1. The R package ‘ggplot2’ was used to visualize the distribution of the moving average of depth-of-coverage, SNP positions, and SNP density over the chromosomes. Furthermore, the moving average of differences in depth-of-coverage (∆depth) between the wild-type and the mutants was calculated. To estimate the 95% confidence interval (q95) and 99% confidence interval (q99), the moving average of depth-of-coverage was randomly extracted from each sample, and ∆depth between the samples was calculated. After this, the operation was repeated 4,000 times, the top 5% of ∆depth was designated as q95, and the top 1% of ∆depth as q99.

To evaluate the depth-of-coverage that was sufficient to detect large deletions induced by gamma irradiation, simulations were conducted by subsampling short reads using seqtk version 1.3-r106 [70]. The moving average of depth-of-coverage and ∆depth over chromosomes was calculated and visualized under depth-of-coverage ranging from 2.5 × to 15 × . Under the 5 × depth-of-coverage conditions, the deletion length was simulated by changing the deletion length to 40, 20, 10, 5, 3, and 1 Mbp in four window size conditions: 1, 3, 5, and 10 Mbp.

To estimate sensitivity and specificity of the ∆depth method under 5 × depth-of-coverage with 1 Mbp window size condition, simulations were conducted by randomly introducing deletions to chromosomes 3A, 3B, and 3D of the “Kitahonami” consensus sequence. The “Kitahonami” consensus sequence was constructed using BCFtools version 1.9 [35] based on the reference genome of CS version 1.0 [8]. The lengths of the introduced deletions for simulation were 1, 10, 100, 300, 500, and 700 kbp, and 1, 5, and 10 Mbp. Short reads were generated from deletion-introduced sequences using BBMap version 37.77 [68]. A deletion was considered to have been detected if the deletion range overlapped by more than 80% with the areas where ∆depth exceeded 95% and 99% confidence intervals using BEDTools version 2.29.2 [71]. After this, the operation was repeated 100 times, and the average sensitivity and sensitivity were calculated. In addition, percentages of true positives and false positives for each length were calculated.

The command lines, packages, and scripts for the above analyses of deletions are described in Additional file 1: Table S3. The R and Python scripts are available from the GitHub repository (https://github.com/ShoyaKomura/WheatGammaIrradiationMutGenomeSeq).

### Marker constructions for deletions and deleted genes

The borders of the large deletion in the PHS mutant of “Kitahonami” were estimated based on the distribution of the moving average of depth-of-coverage. Primers were constructed inside the deletion; two primers were designed outside the deletion. The primers *Pina-D1* and *Pinb-D1*, described by Miki et al. [72], were used to confirm the presence or absence of these genes in the wild-type, the grain hardness mutant, and their F_2_ progenies. The sequence information of primers for markers and genes is listed in Additional file 1: Table S1. The PCR conditions for the markers and genes are as follows: Pre-denaturing at 94 °C for 2 min, 40 cycles of denaturation at 94 °C for 20 s, annealing at 60 °C for 30 s, and extension at 68 °C for 30 s, followed by post-extension at 68 °C for 1 min. For PCR amplification of the co-dominant marker, three primers were mixed before PCR.

## Supplementary Information

**Additional file 1.** 

**Additional file 2.**

**Additional file 3.** 

## Data Availability

The genome sequencing data generated during this study are available in the database of the Komugi Genome Sequencing Program (https://komugigsp.dna.affrc.go.jp/research/download/index.html). The R and Python scripts used in this study are available from the GitHub repository (https://github.com/ShoyaKomura/WheatGammaIrradiationMutGenomeSeq). The plant materials used and/or analyzed during the current study are available from the corresponding author upon reasonable request. We comply with the IUCN policy statement on research involving species at risk of extinction and the Convention on the Trade in Endangered Species of Wild Fauna and Flora. All methods were carried out in accordance with relevant guidelines and regulations in ethics section as plants are used

## Abbreviations

PHS: Pre-harvest sprouting
HA: Hardness
SKCS: Single kernel characterization system
ABA: Abscisic acid
