# Supplementary material for: Genome sequencing-based coverage analyses facilitate high-resolution detection of deletions linked to phenotypes of gamma-irradiated wheat mutants
Source: BMC Genomics. 2022 Feb 9;23:111. doi: 10.1186/s12864-022-08344-8 (PMC8827196; doi:10.1186/s12864-022-08344-8)
Supplement: Supplementary file 1 — Additional file 1. [file 12864_2022_8344_MOESM1_ESM.docx]

**Table S1** Primers for the markers and the genes to detect deletions.

| Target | Primer name | Primer sequences |
| --- | --- | --- |
| 5D-deletion  (*Pina-D1*) | Pina1_F^1^ | CCACCTGCACCAAAACACAC |
|  | Pina1_R^1^ | TATGTTTGCTATAGACACCT |
| 5D-deletion  (*Pinb-D1*) | Pinb1_F^1^ | AATAAAGGGGAGCCTCAACC |
|  | Pinb1_R^1^ | CGAATAGAGGCTATATCATCACCA |
| 2A-deletion | 30579_2A_F | GATTTTGAAGCACACGTTGCCG |
|  | 30579_2A_R | CTCTGCAAATCCAGAGCGACG |
| 4B-deletion | 30579_4B_F | ATTCATTCGACGCCCTTCCTCTAT |
|  | 30579_4B_R | CACAAAAGGGCAACAAATATGTGCA |
| Co-dominant | pre_deletion_primer | ATACACCGTCCACAGTGTTTTGATG |
|  | in_deletion_primer | AATATTGCAGGGAGCTTAAGCGTG |
|  | post_deletion_primer | CATAAGAAGGTTTCGTTCAGTGGCC |
| *Viviparous-B1* | Vp-1B_2F | GATGATGATACTAAAGTAGCTATCGGTTTCTT |
|  | Vp-1B_2R | CCCAAGCCTTGCAAGATTATATCGAC |
|  | Vp-1B_3F | ATATGCATGTTGCGAACTGATCAGTT |
|  | Vp-1B_3R | AAGAACACAGATAGTTCTGCTAGTACGT |
| GRAS transcription factor gene | GRAS-TF_2F | TGGGCCTTGGCCCTTGGAG |
|  | GRAS-TF_2R | TGTTCACATACCTAACTTGCACTGTAC |
|  | GRAS-TF_3F | ACCCTCTCCTTCCCATTCCGTC |
|  | GRAS-TF_3R | CCCAAGAATATCCCCAAAGAGACAAGGAAG |

^1^Miki et al. 2020

**Table S2** Comparisons between putative gamma-irradiated Indel mutations and natural Indel mutations

|  | Wild type vs. 30579  (Hard grain mutant) | Wild-type vs. 28511  (PHS tolerance mutant) | Wild-type vs. CS |
| --- | --- | --- | --- |
| Total | 331 | 271 | 732,510 |
| Exon | 0  (0.000) | 7  (2.583) | 2,456  (0.336) |
| Intron | 9  (2.719) | 10  (3.690) | 26,994  (3.690) |
| UTRs | 6  (1.813) | 0  (0.000) | 6,481  (0.886) |
| Intergenic | 316  (95.468) | 254  (93.727) | 705,922  (96.502) |
|  |  |  |  |
| High impact variants |  |  |  |
| Total | 0  (0.000) | 7  (2.583) | 199  (0.027) |
| Nonsense mutations | 0  (0.000) | 0  (0.000) | 15  (0.002) |
| Start codon lost | 0  (0.000) | 0  (0.000) | 25  (0.003) |
| Stop codon lost | 0  (0.000) | 0  (0.000) | 20  (0.003) |
| Splice sites | 0  (0.000) | 0  (0.000) | 139  (0.019) |

^1^Variant types are defined in SnpEff (Cingolani et al. 2012).

**Table S3** Command lines used for SNP and indel calling

| Process | Command lines |
| --- | --- |
| Alignments | |
| Alignments | bwa index iwgsc_refseqv1.0_all_chromosomes.fasta |
|  | bwa mem -R {read_group} iwgsc_refseqv1.0_all_chromosomes.fasta {R1_paired} {R2_paired} \| samtools view -bF4 > Output.bam |
| Merge of bam files into one bam files | samtools merge merged.bam input1.bam input2.bam input3.bam … |
| Removal of PCR duplicate | samtools sort -n -o merged_sortn.bam merged.bam samtools fixmate -m merged_sortn.bam merged_sortn_fixmate.bam samtools sort -o merged_sortn_fixmate_sort.bam merged_sortn_fixmate.bam samtools markdup -r merged_sortn_fixmate_sort.bam markdup.bam |
| Counting mapped reads and estimating coverage | pileup.sh in=markdup.bam out=mapping_info.tsv |
| Calculation of read depth | samtools depth -a -r <chr1A~chr7D> markdup.bam \| gzip > markdup_depth_<chr1A~chr7D>.tsv.gz |
| Calculation of moving average of read depth | Python script “Calc_MovingAverage.py”  python3 Calc_MovingAverage.py 3000000 1000000 markdup_depth_<chr1A~chr7D>.tsv.gz Average_depth_<chr1A~chr7D>_window3Mbp.tsv  cat Average_depth_*_ window3Mbp.tsv \| sort -k 1,1 -k 2,2n > Average_depth_merged.tsv Python script “Calc_DepthGap.py”  python3 Calc_DepthGap.py WT_Average_depth_merged.tsv Mut_Average_depth_merged.tsv WT_Mut_delta_depth.tsv |
| Visualization of moving average of read depth | Rscript “Plot_MovingAverage.R”  Rscript Plot_MovingAverage.R WT_Average_depth_merged.tsv Mut_Average_depth_merged.tsv WT_Mut_moving_average Rscript “Plot_DepthGap”  Rscript Plot_DepthGap.R WT_Mut_delta_depth.tsv WT_Mut_delta_depth |
| Detection of SNPs and indels between Kitahonami or the mutants and Chinease Spring | |
| Variant calling | bcftools mpileup -a AD -B -Q 0 -A -O u -f iwgsc_refseqv1.0_all_chromosomes.fasta Input.bam \| bcftools view -v indels,snps -O z -o Variant.vcf.gz |
| Filtering of variants  (filtering conditions: Depth >=5, MQ >=40, SNPindex >=0.8) | #SNP  bcftools view -v snps Variant.vcf.gz \| bcftools call -mv - \| bcftools view -i "DP>=5& MQ>=40 & (DP4[2]+DP4[3])/(DP4[0]+DP4[1]+DP4[2]+DP4[3])>=0.8" -O v -o SNPs_filt.vcf - |
|  | #Indel  bcftools view -v indels Variant.vcf.gz \| bcftools call -mv - \| bcftools view -i "DP>=5& MQ>=40& IMF >=0.8" -O v -o Indels_filt.vcf - |
| Detection of SNPs and indels between Kitahonami and the mutants | |
| Obtaining unique variants of each sample | bcftools isec -n -1 -p isec_3sample -c all kitahonami.vcf.gz 28511.vcf.gz 30579.vcf.gz |

**Table S3** -continued.

| Process | Command lines |
| --- | --- |
| Filtering of unique variants  (Filtering conditions: Depth >=5, MQ >=40, SNPindex >=0.8 | #SNP  bcftools view -v snps Unique_variant.vcf.gz \| bcftools call -mv - \| bcftools view -i "DP>=5& MQ>=40& (DP4[2]+DP4[3])/(DP4[0]+DP4[1]+DP4[2]+DP4[3])>=0.8" -O v -o Unique_SNPs_filt.vcf |
|  | #Indels  bcftools view -v indels Unique_variant.vcf.gz \| bcftools call -mv - \| bcftools view -i "DP>=5& MQ>=40& IMF >=0.8" -O v -o Unique_Indels_filt.vcf - |
|  |  |
| Clarifying nucleotides of the other samples at positions of unique variants of the target samples  (Filtering conditions:   1. Read depth of wild type allele > 4 in one of the other samples. 2. Read depth of mutant allele = 0 in both of the other samples. 3. Read depth of mutant allele > 4 in the target sample.   Homozygous mutant alleles in the target sample. | #The filtered variants were merged into “Unique_filt_merged.vcf”.  bcftools concat -O v -a -o Unique_filt_merged.vcf Unique_SNPs_filt.vcf Unique_Indels_filt.vcf  #Creating a multiple mpileup file  #30579  bcftools mpileup -a AD -B -Q 0 -A -O u -R Unique_filt_merged.vcf -f iwgsc_refseqv1.0_all_chromosomes.fasta kitahonami.bam 28511.bam 30579.bam \| bcftools view -v indels,snps -O u \| bcftools call -mv -O v -o 30579_variant_region_multi_sample.vcf  bcftools view -e 'AD[0:0] <5 && AD[1:0] <5 \|\| GT[2]!=”hom” \|\| AD[2:1] <5 \|\| AD[0:1]!=0 \|\| AD[1:1]!=0' -O v -o 30579_variant_region_multi_sample_Depth_GT_filt.vcf 30579_variant_region_multi_sample.vcf  #28511  bcftools mpileup -a AD -B -Q 0 -A -O u -R Unique_filt_merged.vcf -f iwgsc_refseqv1.0_all_chromosomes.fasta kitahonami.bam 30579.bam 28511.bam \| bcftools view -v indels,snps -O u \| bcftools call -mv -O v -o 28511_variant_region_multi_sample.vcf –  bcftools view -e 'AD[0:0] <5 && AD[1:0] <5 \|\| GT[2]!=”hom” \|\| AD[2:1] <5 \|\| AD[0:1]!=0 \|\| AD[1:1]!=0' -O v -o 28511_variant_region_multi_sample_Depth_GT_filt.vcf 28511_variant_region_multi_sample.vcf |
| SnpEff | snpeff eff -spliceRegionExonSize 0 -spliceRegionIntronMin 0 -spliceRegionIntronMax 0 -ud 0 Chinese_spring variant_region_multi_sample_Depth_GT_filt.vcf > variant_region_multi_sample_Depth_GT_filt.snpeff |
| Counting SNPs in each window | Python script “Count_SNPs_per_window.py”  python3 Count_SNPs_per_window.py 10000000 SNPs.vcf |
| Visualization of SNP  positions | R script “Plot_SNP_density.R”  R script Plot_SNP_density.R Number_of_SNPs.tsv output_directory  Python script “Vcf2SNP.py”  python3 Vcf2SNP.py SNPs.vcf SNP_position.tsv  R script “Plot_SNP_position.R”  Rscript Plot_SNP_position.R SNP_position.tsv SNP_position_image |

**Table S3** -continued.

| Process | Command lines |
| --- | --- |
| Simulations | |
| Subsampling of short reads | seqtk sample -s {seeds} <raw_fastq.gz> {output reads ratio} \| gzip > subsampled.fastq.gz  output reads ratio = target average of depth-of-coverage/average depth-of-coverage of raw read data |
| Adjustment of  deletion region | Python script “deletion_control.py”  python3 deletion_dontrol.py 28511_depth_chr3B.tsv.gz WT_depth_chr3B.tsv.gz {deletion size (Mbp)} |
| Introduction of deletions | Python script “Make_deletion.py”  python3 Make_deletion.py {sequence.fasta} deletion_sequence.fasta  #generate short reads from deletion introduced sequence  randomreads.sh ref= deletion_sequence.fasta out1=random_1.fastq out2=random_2.fastq length=150 coverage=5 paired=t replacenoref=t illuminanames=t  #Arrange detected region  Python script “Arange_deletion_region.py”  python3 Arange_deletion_region.py {step_size} filtered_delta_depth.tsv filtered_delta_depth_arranged.tsv  #check overlaps  bedtools intersect -u -e -f 0.8 -F 0.8 -a introduced_deletions.bed -b detected_deletions.bed > True_positive_deletions.bed |
| Construction of  ‘Kitahonami’ consensus  sequence | #normalization of variants  bcftools norm -f iwgsc_refseqv1.0_all_chromosomes.fasta -O z -o norm.vcf.gz Variants_to_CS.vcf.gz  bcftools filter –IndelGap 5 -O z -o norm_filtIndel.vcf.gz norm.vcf.gz  bcftools norm -f iwgsc_refseqv1.0_all_chromosomes.fasta -O z -m +any -o norm_filtIndel_norm.vcf.gz norm_filtIndel.vcf.gz  #make consensus sequence  cat iwgsc_refseqv1.0_all_chromosomes.fasta \| bcftools consensus -H 1 norm_filtIndel_norm.vcf.gz > Kitahonami_consensus.fasta |
