# Supplementary material for: Genome sequencing-based coverage analyses facilitate high-resolution detection of deletions linked to phenotypes of gamma-irradiated wheat mutants
Source: BMC Genomics. 2022 Feb 9;23:111. doi: 10.1186/s12864-022-08344-8 (PMC8827196; doi:10.1186/s12864-022-08344-8)
Supplement: Supplementary file 2 — Additional file 2. [file 12864_2022_8344_MOESM2_ESM.pdf]

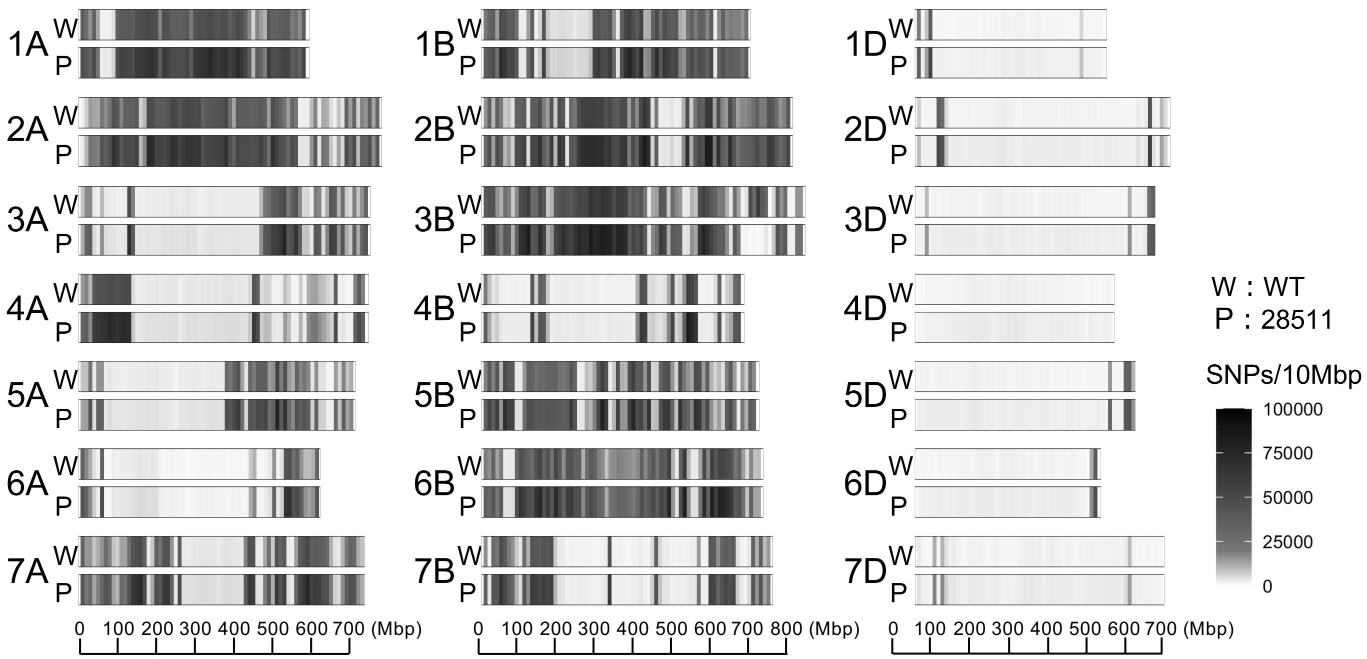

Fig. S1 Distribution of SNPs of wheat cultivars "Kitahonami" and the PHS mutant "28511" along the chromosomes.

SNP density was estimated by counting the number of SNPs per 10 Mb. The SNP density is displayed as a gradation color from white to black. The higher the density, the blacker it is.

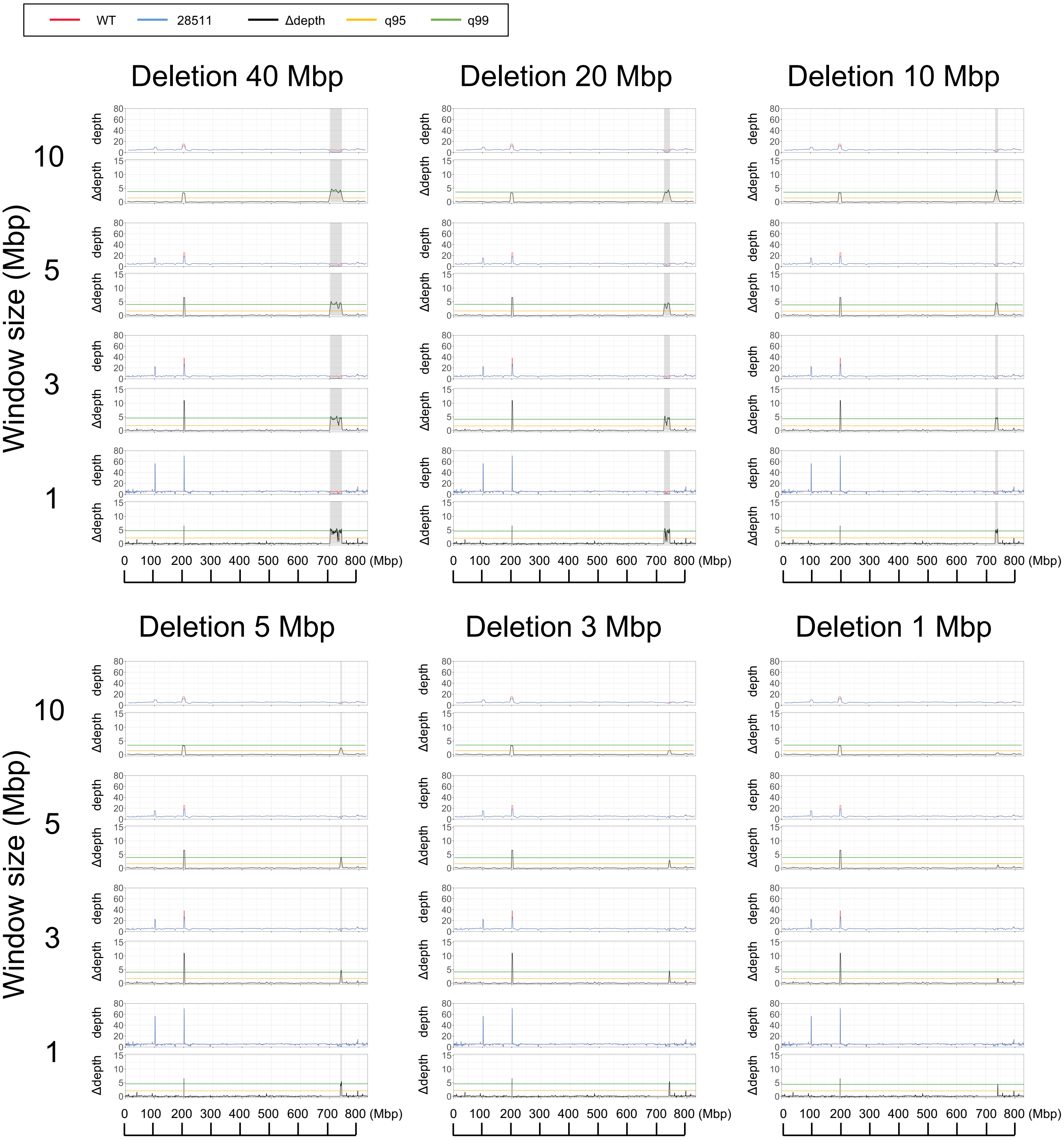

Fig. S2 The tests of deletion detection power under different window sizes and different deletion sizes.

Distribution of the sliding window average for the depth-of-coverage and the differences in depth-of-coverage (Δdepth) between wild-type "Kitahonami" (WT) and the PHS-tolerant mutant "28511" under four window size conditions (1 Mbp, 3 Mbp, 5 Mbp, and 10 Mbp) and six deletion sizes (1, 3, 5, 10, 20, and 40 Mbp), were simulated.

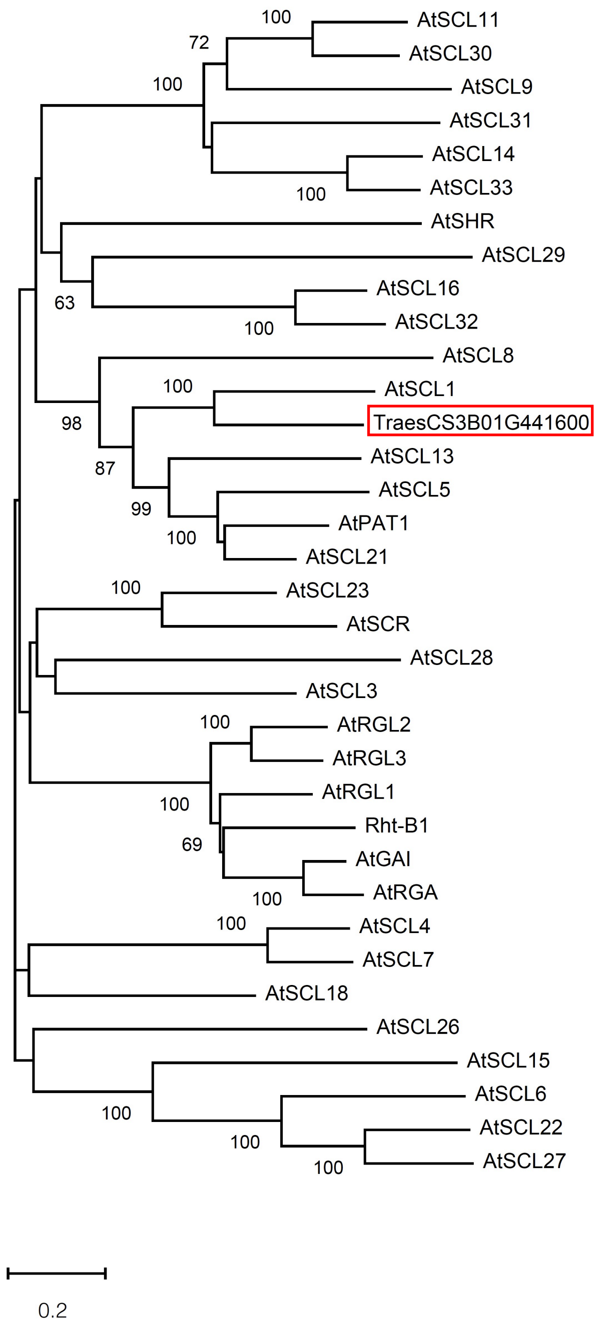

Fig. S3 Evolutionary relationship in GRAS transcription factors.

A neighbor-joining tree of GRAS family transcription factors of *Arabidopsis thaliana* [50] and two wheat GRAS family transcription factors, TraesCS3B01G441600 (in the red rectangle) and Rht-B1 (Thomas 2017) is shown. Bootstrap values in 1,000 replications are shown for each branch. Amino acid sequence alignments were conducted using ClustalW in the MEGA X software (Kumar et al. 2018). A neighbor-joining tree was constructed using MEGA X. The Poisson model was used to estimate distances for the tree.

Thomas SG. Novel *Rht-1* dwarfing genes: tools for wheat breeding and dissecting the function of DELLA proteins. J Exp Bot. 2017;6

Kumar S, Stecher G, Li M, Knyaz C, Tamura K. MEGA X: molecular evolutionary genetics analysis across computing platforms. Mol Biol Evol. 2018;35:1547–9.
