# Supplementary figures and images for: Genome sequencing-based coverage analyses facilitate high-resolution detection of deletions linked to phenotypes of gamma-irradiated wheat mutants
Source: BMC Genomics. 2022 Feb 9;23:111. doi: 10.1186/s12864-022-08344-8 (PMC8827196; doi:10.1186/s12864-022-08344-8)

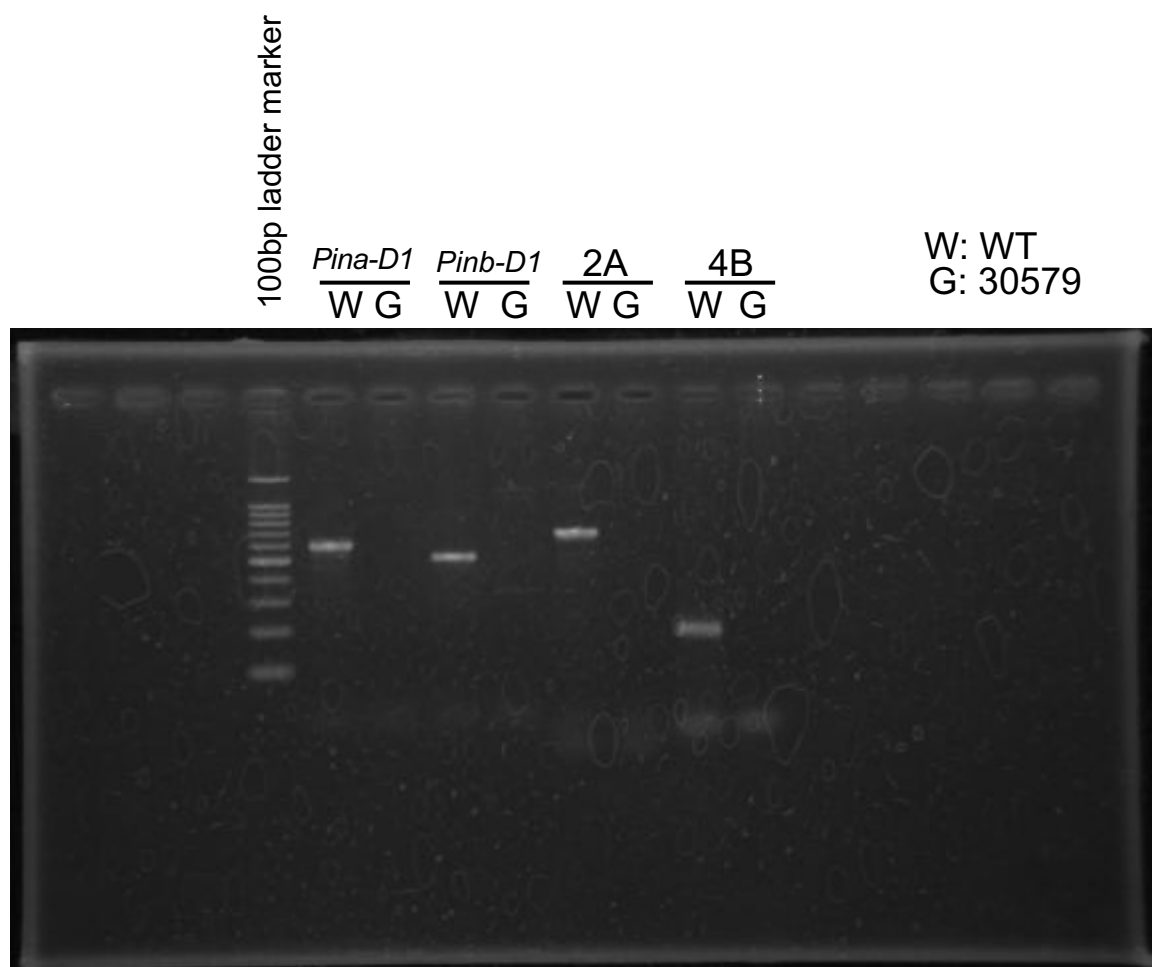

**Fig. S4** The full gel image of Fig. 4b

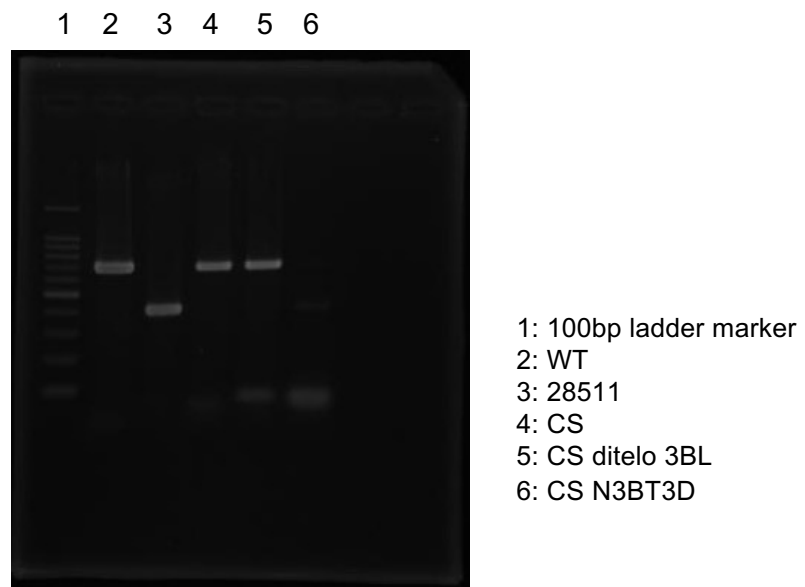

**Fig. S5** The full gel image of Fig. 7b

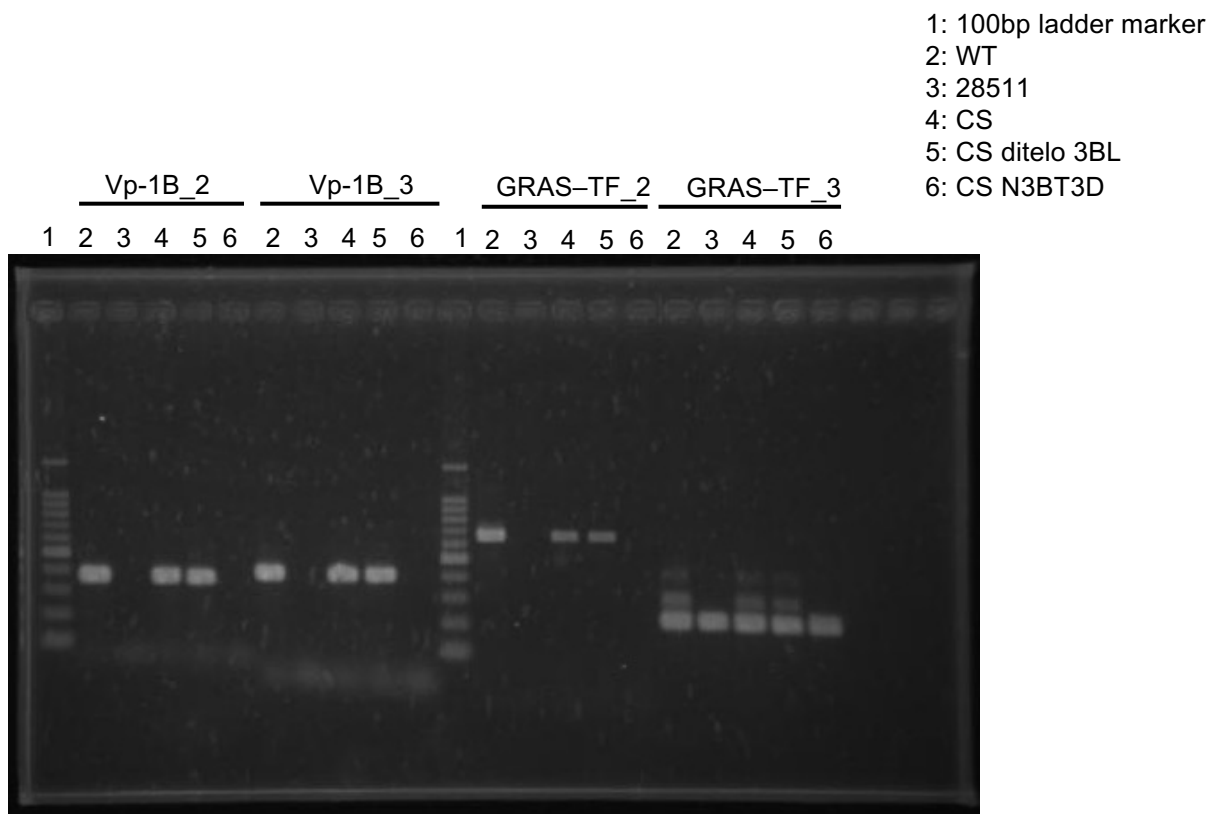

**Fig. S6** The full gel image of Fig. 8b

Supplement: Supplementary file 3 — Additional file 3. [file 12864_2022_8344_MOESM3_ESM.pdf]
